# Barriers to identifying and obtaining CME: a national survey of physicians, nurse practitioners and physician assistants

**DOI:** 10.1186/s12909-021-02595-x

**Published:** 2021-03-19

**Authors:** Maureen O’Brien Pott, Anissa S. Blanshan, Kelly M. Huneke, Barbara L. Baasch Thomas, David A. Cook

**Affiliations:** 1 Planning Services, Mayo Clinic, Rochester, MN USA; 2 Medical Professionals Marketing, Mayo Clinic, Rochester, MN USA; 3 Mayo Clinic School of Continuous Professional Development, Mayo Clinic College of Medicine and Science, Rochester, MN USA; 4 Office of Applied Scholarship and Education Science, Mayo Clinic College of Medicine and Science, Rochester, MN USA; 5 Division of General Internal Medicine, Mayo Clinic, Rochester, MN USA

**Keywords:** Education, medical, continuing, Credentialing, Licensure, medical, Marketing, Workplace learning

## Abstract

**Background:**

CPD educators and CME providers would benefit from further insight regarding barriers and supports in obtaining CME, including sources of information about CME. To address this gap, we sought to explore challenges that clinicians encounter as they seek CME, and time and monetary support allotted for CME.

**Methods:**

In August 2018, we surveyed licensed US clinicians (physicians, nurse practitioners, and physician assistants), sampling 100 respondents each of family medicine physicians, internal medicine and hospitalist physicians, medicine specialist physicians, nurse practitioners, and physician assistants (1895 invited, 500 [26.3%] responded). The Internet-based questionnaire addressed barriers to obtaining CME, sources of CME information, and time and monetary support for CME.

**Results:**

The most often-selected barriers were expense (338/500 [68%]) and travel time (*N* = 286 [57%]). The source of information about CME activities most commonly selected was online search (*N* = 348 [70%]). Direct email, professional associations, direct mail, and journals were also each selected by > 50% of respondents. Most respondents reported receiving 1–6 days (*N* = 301 [60%]) and $1000–$5000 (*n* = 263 [53%]) per year to use in CME activities. Most (> 70%) also reported no change in time or monetary support over the past 24 months. We found few significant differences in responses across clinician type or age group. In open-ended responses, respondents suggested eight ways to enhance CME: optimize location, reduce cost, publicize effectively, offer more courses and content, allow flexibility, ensure accessibility, make content clinically relevant, and encourage application.

**Conclusions:**

Clinicians report that expense and travel time are the biggest barriers to CME. Time and money support is limited, and not increasing. Online search and email are the most frequently-used sources of information about CME. Those who organize and market CME should explore options that reduce barriers of time and money, and creatively use online tools to publicize new offerings.

**Supplementary Information:**

The online version contains supplementary material available at 10.1186/s12909-021-02595-x.

## Background

All health professionals engage to varying degrees in continuous professional development (CPD) – a lifelong process of “improv[ing their] professional knowledge, skills, or performance.” [1] Continuing medical education (CME) is a subset of CPD that awards credit for designated activities. Most clinicians (including physicians, nurse practitioners, and physician assistants) must participate regularly in CME to maintain licensure. Problems have been identified in the way CME is delivered, financed, regulated, and evaluated [2–6], leading to strategic changes in CME delivery, evaluation, and regulation [7–9]. Less well documented, however, are the barriers and challenges (“pain points”) that individual clinicians encounter as they try to meet their CME requirements, and the sources of information they use in identifying CME opportunities.

Most studies examining barriers to CPD are over a decade old [10–14], and given changes in both clinical practice and CME infrastructure their findings may be of limited contemporary relevance. A recent survey of US physicians found that time and money were the top barriers [1, 15], but this study did not involve nonphysicians nor did it explore how clinicians’ CME and CPD activities are financially supported. Another recent survey of physicians, general practice nurses, and pharmacists in Scotland [16] reported the availability of protected time for CME, but did not address other potential barriers or supports. Although prior surveys suggest that finding relevant CPD is a barrier [1, 10, 17, 18], we are not aware of any contemporary research contrasting sources by which clinicians learn about CME opportunities.

CPD educators and CME providers would benefit from further insight regarding barriers and supports in obtaining CME, and sources clinicians use to identify CME offerings. To address this gap, we conducted a national survey of US physicians, nurse practitioners, and physician assistants, addressing questions:

1. What challenges do clinicians encounter as they seek CME, and what support (time and money) do they receive for CME?
2. From what sources of information do clinicians find out about CME opportunities?
3. How do these responses vary by clinician type, specialty, and age?
4. What suggestions do clinicians have for improving CME?

## Methods

### Overview

We surveyed licensed US clinicians (physicians, nurse practitioners, and physician assistants) using an Internet-based questionnaire that addressed pain points in obtaining CME, sources of information regarding CME, and level of support for CME in terms of time and money. Another paper using other questions from this survey has been published, addressing how clinicians select among available CME modalities [19].

### Sampling and human subjects

Clinicians were eligible if they had completed a CME activity through any provider in the preceding 24 months. Clinicians employed by our institution or a closely affiliated institution were excluded. We used stratified random sampling to select clinicians from an external vendor database (Dynata), and invited participants until we achieved our target sample of 100 respondents from each of five clinician types: family medicine physicians, internal medicine and hospitalist physicians, physicians in other internal medicine subspecialties, nurse practitioners (all medical specialties), and physician assistants (all medical specialties). This sample was selected for practical reasons, namely that the research team is responsible for CME programming for this audience. We offered respondents a modest incentive (a gift card, charitable contribution, or similar product, valued at most $44). The study was deemed exempt by the Mayo Clinic Institutional Review Board.

### Instrument

A group of administrators and researchers with experience in CME, including specific expertise in CME operations and marketing, developed the survey questionnaire. We identified relevant issues by reviewing prior internal (unpublished) and published surveys [1, 10, 14], from personal experience, and through discussions with clinicians, CME course directors, operations managers, and marketing analysts. This report presents responses for a subset of questions from the full survey, including items regarding barriers in obtaining CME, sources of information when looking for CME offerings, and time and monetary support for CME. Items used checklists or ordinal response options (see Results and the e-Box for specific wording). We also asked two open-ended questions about “ways [our institution] can enhance their [live, in-person *or* online] educational courses?” The survey questions are provided verbatim in the e-Box.

### Survey administration

Survey administration was conducted via email from August 7 to August 14, 2018 by Endeavor Management Consulting. Waves of email invitations were sent each day, with one reminder on August 13. Each email contained an individually-tracked link to an Internet-based questionnaire hosted on Qualtrics (www.qualtrics.com). The survey closed after achieving the target response (*N* = 100) for each clinician subgroup. All responses were anonymous.

### Analyses

We compared demographics of respondents and nonrespondents using chi-squared. For most outcomes (those with an approximately normal distribution), we used ANOVA to explore differences in across subgroups (clinician specialty and age), and used Tukey’s test to contrast subgroups for statistically significant models. For questions about time and money allotment we used the Kruskall-Wallis test. All analyses were performed using SPSS v25. Given the large sample size and the large number of independent statistical tests, we used *p* < .01 as the threshold of statistical significance.

Two authors (AB, KH) iteratively and independently coded responses to the two open-ended questions, to inductively identify key themes. They then worked together to refine the list of themes into a concise list of potential actions that might mitigate barriers to CME.

## Results

To achieve our target of 500 responses (100 per clinician subgroup) we invited 1895 clinicians (overall response rate, 26.3%); namely 975 family medicine and internal medicine/hospitalists (21% response), 199 specialists (50% response), and 721 nurse practitioners and physician assistants (28% response). Respondents and non-respondents were similar in gender, age, and geographical region (*p* ≥ 0.04; see Table 1).

**Table 1:** Characteristics of Respondents and Non-respondents

Domain	Subdomain	Respondents No. (%); ***N*** = 500	Non-respondents No. (%); ***N*** = 1395	P
Gender	Female	251 (50%)	742 (53%)	.25
	Male	249 (50%)	653 (47%)	
Region of USA^a^	Northeast	140 (28%)	330 (24%)	.04
	Midwest	95 (19%)	292 (21%)	
	South	146 (30%)	491 (35%)	
	West	111 (23%)	279 (20%)	
Age (years)^b^	< 40	92 (24%)	189 (27%)	.59
	40–49	123 (33%)	203 (29%)	
	50–59	87 (23%)	161 (23%)	
	≥60	74 (20%)	147 (21%)	

^a^ *N* = 392 and 1392 for region, for respondents and non-respondents respectively, due to incomplete data in database

^b^ *N* = 376 and 700 for age, for respondents and non-respondents respectively, due to missing responses

### Barriers to CME

When asked to select the most important “pain point” from a checklist, expense (selected by 338/500 [68%] respondents) was the most frequently selected, followed by travel time (*N* = 286 [57%]), discovering/searching for appropriate CME offerings (*N* = 143 [29%]), and topic not applicable to daily practice (*N* = 133 [27%]) (see Table 2).

**Table 2:** Barriers to Obtaining CME, by Clinician Type

Barrier	All: No. (%); N = 500	FM: No.; ***N*** = 100	IM/H: No.; N = 100	Spec.: No.; N = 100	NP: No.; N = 100	PA: No.; N = 100	p
Expense	338 (67.6%)	67	65	60	77	69	.13
Travel time	286 (57.2%)	57	55	63	51	60	.48
Discovering/searching for appropriate CME offerings	143 (28.6%)	28	29	27	34	25	.70
Not applicable to daily practice	133 (26.6%)	25	21	30	25	32	.41
Ability to easily track CME credits earned	59 (11.8%)	16	12	11	14	6	.25

Responses were selected from a checklist, in response to the question: “What are the biggest gaps/pain points in obtaining CME offerings? [check all that apply]”

*Abbreviations*: *FM* Family Medicine, *IM/H* Internal Medicine / Hospitalist, *Spec.* Internal Medicine Specialist, *NP* Nurse Practitioner, *PA* Physician Assistant

There were no statistically significant differences in barriers across clinician types. There was one statistically significant difference by age groups, with those ≥60 (61/74 [82%]) more likely to select expense than those < 40 (55/92 [60%]) (*p* = .01; see e-Table 1).

### Sources of information

When asked to identify where they get information about CME activities, the source of information most commonly selected was online search (*N* = 348 [70%]). Other sources selected by > 50% of respondents included email, professional associations, direct mail brochures, and journals. Mentors (4%) and supervisors (3%) were identified least often. See Table 3 for details on additional sources.

**Table 3:** Sources of Information about CME Courses, by Clinician Type

Information Source	All: No. (%); ***N*** = 500	FM: No.; ***N*** = 100	IM/H: No.; N = 100	Spec.: No.; ***N*** = 100	NP: No.; ***N*** = 100	PA: No.; ***N*** = 100	p
Online	348 (69.6%)	70	64	68	69	77	.38
Direct email communication	296 (59.2%)	68	50	62	63	53	.06
Professional associations	293 (58.6%)	61	47^a^	50	70^a^	65	.003
Brochures via direct mail	263 (52.6%)	57	39^a^	52	71^a^	44^a^	<.001
Journals	260 (52.0%)	45	50	56	57	52	.44
Peers/word of mouth	234 (46.8%)	41	42	42	52	57	.08
Healthcare organizations	165 (33.0%)	32	29	26^a^	48^a^	30	.009
Medical societies	163 (32.6%)	39	34	37	26	27	.18
Brochures via in-person handout	104 (20.8%)	15^a^	16^a^	15^a^	39^a^	19^a^	<.001
Doximity	54 (10.8%)	11	9	16	9	9	.43
Physician liaison	27 (5.4%)	4	6	9	5	3	.39
Online chat forums	24 (4.8%)	4	5	6	5	4	.96
A mentor	21 (4.2%)	6	5	5	3	2	.61
My supervisor	15 (3.0%)	3	2	1	6	3	.31

Responses were selected from a checklist, in response to the question: “How do you find out about CME courses?”

*Abbreviations*: *FM* Family Medicine, *IM/H* Internal Medicine / Hospitalist, *Spec*. Internal Medicine Specialist, *NP* Nurse Practitioner, *PA* Physician Assistant

^a^ Subgroup responses are statistically significantly different from one another using Tukey’s test (p < .01); in each instance, nurse practitioners’ selections were more frequent than those of the other indicated clinician type(s)

We found statistically significant differences (*p* < .01) across clinician types for four (of 14) information sources; in each instance, nurse practitioners were more likely than another clinician type to select that information source. Specifically, nurse practitioners were more likely (70/100) than internal medicine physicians (47/100) to select professional associations as an information source; more likely (71/100) than physician assistants (44/100) and internal medicine physicians (39/100) to select direct mail brochures; more likely (48/100) than specialists (26/100) to select healthcare organizations; and more likely (39/100) than all other groups (all ≤19%) to select in-person handouts.

We found statistically significant differences (*p* < .01) by age group for three information sources. Specifically, those 50–59 years old were more likely (43/87 [49%]) than those 40–49 (32/123 [26%]) to select healthcare organizations as an information source; those 50–59 were more likely (59/87[68%]) than those < 40 (40/92[44%]) to select direct mail brochures; and those ≥60 (56/74[76%]) were more likely than both those < 40 (46/92[50.0%]) and those 40–49 (64/123[52%]) to select email communication.

### Time and monetary support

We asked participants to indicate how much time and money they were allotted to support CME activities, and how much this had changed over the past 24 months (see Table 4). The most frequently cited number of days allotted for CME was 1–6 days per year (301/500; 60%). The most frequently cited dollar amount for CME support was USD $1000 - $5000 per year (263/500 [53%]). No statistically significant differences (*p* > .01) in current time or monetary support were identified by clinician type or age (see Table 4 and e-Table 3).

**Table 4:** Time and Monetary Support Available for CME, by Clinician Type

Domain	Response	All: No. (%); ***N*** = 500	FM: No.; ***N*** = 100	IM/H: No.;***N*** = 100	Spec.: No.;***N*** = 100	NP: No.; ***N*** = 100	PA: No.; ***N*** = 100	p
Time allotment	0 days	59 (11.8%)	10	10	17	11	11	.03
	1–6 days	301 (60.2%)	52	55	44	77	72	
	≥7 days	121 (24.2%)	30	29	34	12	16	
	Irrelevant^a^	19 (3.8%)	7	6	5	0	1	
Change in time allotment	Less	79 (15.8%)	17	14	16	17	15	.33
	No change	374 (74.8%)	73	71	80	77	73	
	More	47 (9.4%)	10	15	4	6	12	
Monetary allowance	<$1000/ none	219 (43.8%)	40	50	49	47	33	.30
	$1000 - $5000	263 (52.6%)	55	48	41	52	67	
	>$5000	18 (3.6%)	5	2	10	1	0	
Change in monetary allowance	Less	78 (15.6%)	11	11	11	17	18	.21
	No change	358 (71.6%)	68	71	71	75	73	
	More	45 (9.0%)	10	14	7	7	7	
	No support	29 (5.8%)	11	4	11	1	2	

Questions asked about current time or monetary support from their practice for CME activities each year, and how the amount of time/monetary support had changed over the past 24 months

*Abbreviations*: *FM* Family Medicine, *IM/H* Internal Medicine / Hospitalist, *Spec.* Internal Medicine Specialist, *NP* Nurse Practitioner, *PA* Physician Assistant

^a^ Indicates clinicians who would “Not [be] willing to be away from my practice”

The vast majority of respondents (> 70%) indicated no change over the past 24 months in time or monetary support (see Table 4). Less than 10% indicated that support for CME activities has increased in this time period, and about 14% indicate that support has actually decreased (see Table 4). Again, we found no statistically significant differences by clinician type or age groups (Table 4 and e-Table 3).

### Opportunities for enhancing CME

We inductively and iteratively analyzed the 773 free-text responses to two questions asking for suggestions to enhance in-person and online CME. From this analysis we identified eight proposed enhancements, many of which will help mitigate barriers noted above. See Table 5 for additional quotes in support of each enhancement.

**Table 5:** Suggested Actions to Enhance CME Activities, and Supporting Quotes

Suggested action^**a**^	Quotes
Optimize location	• Good locations and maybe include families in some activities. • Have it in multiple cities. • Hold them around the country in various locations.
Reduce cost	• Create inexpensive offerings in the Pacific NW. • Keep expenses under control. • Lower the cost for meeting & for hotel costs.
Publicize effectively	• Better knowledge of when courses are. • Better notification, I am largely unaware of courses offered but maybe I am not on the proper mail/email list. • Promoting better awareness of these courses and the benefits associated. • I just need to know that they are happening. • Publicize them more so more providers will be aware. • Advertising more on topics presented and when. • Send more brochures and advertise more in professional magazines.
Offer more	• Greater availability in a variety of locations. • Higher volume of courses offered. • Have at destination locations and content that is applicable to daily practice problems along with some new insights, etc. • More access via email and internet.
Allow flexibility	• Make at convenient times, after office hours. • Make more Category 1 and allow flexible time for participation. • More available times and dates. • Short courses, no post testing. • Keep flexible times. • Offer more weekend courses, too busy during the week.
Ease of use and accessibility	• Their system can be difficult to access. It needs to be more user-friendly. • Ease of use (time, availability, at user’s pace).
Make it clinically relevant	• A lot of times the talks are a lot about graphs and fluff instead of point blank how it can affect your clinical practice on a daily basis. • Good engaging speakers with clinical pearls.
Encourage application	• More interactive. • More visual aids.

^a^ Suggestions derived from inductive analysis of 773 free-text comments

#### Optimize location

Location matters, whether it is conveniently nearby or in a “desirable destination.” One respondent mentioned “having [courses] in multiple cities around the country and at locations near the airport.” Another suggested having “a variety of specialty specific courses in desirable location with some free time,” while another mentioned having “15 credits per day x 2 days in desirable location.”

#### Reduce cost

Clinicians are willing to pay for CME, but would like it to cost less, whether in-person or online. Many respondents specifically used phrases such as “cost,” “cost effective,” “decrease cost,” and “inexpensive offerings.” It is not just the cost of registration, but the overall cost to attend; as one respondent suggested, “Lower the cost for meeting and hotel costs.” Notably, one respondent expressed concern that their “CME allowance has not changed in 30 years, but the cost of courses has gone up.”

#### Publicize effectively

Clinicians want to know about their options and prefer that information be disseminated well in advance. As one respondent noted, “I just need to know when they are happening.” Another said, “Find a way to reach those who could use the CME.” Other specific suggestions included: “better advertisement of availability,” “better outreach,” and “better publicity of the courses.”

#### Offer more

Clinicians want more options for CME. Respondents indicated they would like more CME content, addressing a greater variety of topics, offered in higher volumes (“more offerings,” “more locations,” “more variety of topics,” and “more courses available”). One person requested, “Gear them to nurse practitioners as well as MDs.”

#### Allow flexibility

Clinicians need flexibility in course structure and scheduling, and this is true for both in-person and online courses. For in-person courses, participants commented “convenient time on weekend,” “flexibility in dates and length of course,” “vary the time of events- I like 5 pm Pacific; Sat & Sundays ok with me.” There was also a suggestion that the “days should be shorter so that they could enjoy the location.”

For online courses, participants suggested “allowing more up to date practices and ability to go back if topic is misunderstood rather than feeling rushed,” “available 24 hours 7 days,” “better times” and “flexible times and do at your own pace.”

#### Ensure ease of use and accessibility

Ease of use and accessibility are critical to customer satisfaction and success. Multiple respondents mentioned “access,” “availability” and “having resources available at the user’s pace.” They specifically called for “wider availability of topics,” “easy availability of numerous topics at a reasonable price,” “time availability,” and “wider availability [of] options.”

#### Make it clinically relevant

Course content needs to be relevant to daily practice (“relevant topics and how it will be used in practice”). This involves both selecting clinically-relevant topics for a course or session, and making content within a session clinically relevant. Respondents requested speakers who shared “clinical pearls” or “pitfalls,” and encouraged a clear link to how the topic can affect their clinical practice using phrases such as “more topics used in my clinic,” “more family practice topics,” and “more specific courses for my sub-specialty.” A commonly-suggested approach was to use “plenty of clinical scenarios.”

#### Encourage application

Clinicians recognize the need for opportunities to apply what they have learned. Several respondents requested that courses be “more hands-on.” Others endorsed activities that promote application of learning to authentic cases, such as having “lectures … structured for daily practice routines supplemented with challenging cases [and] pitfalls for each topic.”

## Discussion

This national survey of 500 US physicians, nurse practitioners, and physician assistants found that expense and travel time are the biggest barriers. Clinicians most often rely on online search, direct email communications, professional associations, mailed brochures, and journal advertisements to find out about CME opportunities. Time and monetary support for CME has not changed in the past 24 months. These findings were generally similar across clinician types and age groups. Open-ended responses highlight several opportunities for improvements in CME delivery, including optimizing location, lowering costs, publicizing courses more effectively, providing opportunities for application, and making content clinically relevant.

### Limitations

Our study has several limitations that allow the possibility that our findings do not represent the larger population of US clinicians. The email administration format might have preferentially selected for individuals more comfortable with online and email communication, and the short response period may have given preference to early responders. The relatively low overall response rate also raises the possibility of a suboptimally-representative sample, although respondents and nonrespondents were similar in observed demographics. Additionally, we did not invite physicians in non-medicine specialties. Although the survey developers had extensive experience in CME and marketing, the choice of survey topics and final wording of survey items represents another potential limitation. Strengths include the large national sample and the inclusion of nurse practitioners, physician assistants, and physicians in various medicine specialties.

### Integration with prior work

Our findings elaborate on prior surveys about CME [1, 10–15, 20] by focusing on barriers, and time and monetary supports (and how these have changed). This study also prioritizes the channels by which clinicians find out about CME opportunities, and proposes enhancements that may mitigate some barriers.

### Implications for practice and future research

We highlight several implications of our work. First, CME providers seeking to publicize an upcoming event will find our prioritized list of information sources (i.e., marketing channels) useful. While it is perhaps unsurprising that user-initiated Internet search led this list, this finding underscores the need for CME providers to maintain a strong online presence. Search engine optimization [21, 22] can enhance the likelihood that a given website will be ranked highly in a user-initiated “organic” search (search results comprised of unadvertised, unpaid links). The preference for email aligns with findings from a survey conducted by a commercial vendor (88% preference for CME information email vs. direct mail, journals, or social media) [23]. Although search engine optimization and strategic email marketing are well-established approaches in consumer marketing, they have received little dedicated study in CME. We note anecdotally that our institution has had mixed experiences with direct email marketing for CME; whether this represents idiosyncrasies related to our specific email strategy, or a cautionary note regarding this modality for CME generally, remains unexplored.

Second, most clinicians have limited resources (less than one week and $5000 per year) allotted for CME, and this allotment is not changing for most clinicians. As one clinician pointed out, unchanged monetary support translates to an overall reduction in purchasing power over time. Perhaps more importantly, the modest time allotment highlights that CME cannot always require travel. Previous research suggests that overall cost and topical relevance are the most influential factors in selecting CME opportunities requiring travel [15]. Online CME has the potential to overcome barriers of both out-of-pocket cost and time required for travel [24], could exploit several of the opportunities noted above (e.g., flexibility, accessibility, and volume), and is now widely accepted across all age groups and clinician types [1, 19, 20]. However, we remind readers that the instructional designs, delivery approaches, and implementation strategies that foster effective online learning typically differ from those used in face-to-face instruction (thus requiring unique faculty skillsets, innovative tools, and novel course designs) [25, 26], and that the development cost of online learning activities can be high [27].

Third, our data indicate that nurse practitioners tended to select a variety of information sources with higher frequency than other clinician types. These findings, together with the quote from one respondent, could indicate that nurse practitioners feel obligated to cast a broader net to find CME courses relevant to their needs. They could also simply reflect different response patterns for this group. Pending further study, our findings suggest that nurse practitioners may benefit from more, and more publicity about, CME options that target their needs.

Fourth, the CPD landscape has been transformed by the COVID-19 pandemic, with online learning filling the void created by curtailed face-to-face offerings. Now that CME providers and consumers have experienced the advantages of distance learning, it seems almost certain that some degree of transformation will endure. This in turn may affect the salience of some barriers (e.g., cost, time, flexibility). Repeating a survey such as ours in the post-COVID era may prove insightful.

Finally, our findings support the advantage of a strong infrastructure to support CME as an enterprise. Six of the suggestions to enhance CME (location, cost, publicity, clinical relevance [of the course], and quantity and accessibility of offerings) address issues that extend beyond the teaching within a session, and require operational decisions months or years in advance. While the CME enterprise is often criticized [2–6], our findings suggest that it is nonetheless important. Reduced costs, increased quantity of offerings, and enhanced publicity are all more likely to be achieved through experienced CME providers. This underscores the need to both support and regulate CME providers, as they in turn support clinicians in their pursuit of ongoing professional development.

## Conclusions

Clinicians report that expense and travel time are the biggest barriers to CME. Time and money support is limited, and not increasing. Online search and email are the most frequently-used sources of information about CME. Those who organize and market CME should explore options that reduce barriers of time and money, and creatively use online tools to publicize new offerings.

## Supplementary Information

**Additional file 1 Online Supplemental Box and Tables: e-Box**. Verbatim Items from Survey Questionnaire. **e-Table 1**. Barriers in Obtaining CME, by Age Group. **e-Table 2**. Sources of Information about CME Courses, by Age Group. **e-Table 3**. Time and monetary support available for CME, by Age Group.

## Data Availability

All data generated or analysed during this study are included in this published article and its supplementary information files.

## Abbreviations

CPD: Continuous professional development
CME: Continuing medical education
