# Supplementary material for: Barriers to identifying and obtaining CME: a national survey of physicians, nurse practitioners and physician assistants
Source: BMC Med Educ. 2021 Mar 19;21:168. doi: 10.1186/s12909-021-02595-x (PMC7975233; doi:10.1186/s12909-021-02595-x)
Supplement: Supplementary file 1 — Additional file 1 Online Supplemental Box and Tables: e-Box. Verbatim Items from Survey Questionnaire. e-Table 1. Barriers in Obtaining CME, by Age Group. e-Table 2. Sources of Information about CME Courses, by Age Group. e-Table 3. Time and monetary support available for CME, by Age Group. [file 12909_2021_2595_MOESM1_ESM.docx]

Online-only supplemental materials

# e-Box. Verbatim Items from Survey Questionnaire

What are the biggest gaps/pain points in obtaining CME offerings? [check all that apply]

- Expense
- Travel time
- Not applicable to daily practice
- Ability to easily track CME credits earned
- Discovering/searching for appropriate CME offerings

How do you find out about CME courses? [check all that apply]

- Journals
- Brochures via direct mail
- Brochures via in-person handout (e.g. at a conference or training session))
- Online
- Direct email communication
- Doximity
- Online chat forums
- Peers/word of mouth
- My supervisor
- A mentor
- Physician liaison
- Healthcare organizations
- Professional associations
- Medical societies
- Other (Please specify)

How much time does your practice allot per year to attend CME-related activities?

- 0 days
- 1 -3 days
- 4 -6 days
- 7-9 days
- 10 or more days
- Not willing to be away from my practice

Which of the following best describes how the amount of time allocated by your practice for CME has changed over the past 24 months?

- A lot less time allocated
- A little less time allocated
- No change
- A little more time allocated
- A lot more time allocated

Do you receive an annual monetary allowance for CME courses/activities? [yes/no]

[If "Yes" to above] How much annual monetary allowance do you receive?

- Less than $1,000
- $1,000-$5,000
- $5,001-$10,000
- $10,001-$15,000
- More than $15,000

Which of the following best describes how the monetary support allocated by your practice for CME has changed over the past 24 months?

- A lot more money
- A little more money
- No change
- A little less money
- A lot less money
- Money is no longer provided for continuing education

In what ways can Mayo Clinic enhance their live, in-person educational courses? (OPEN END)

In what ways can Mayo Clinic enhance their online educational courses? (OPEN END)

# e-Table 1. Barriers in Obtaining CME, by Age Group

| **Barrier** | **All: No. (%);**  **N=500** | **<40: No. (%);**  **N=92** | **40-49: No. (%);**  **N=123** | **50-59: No. (%);**  **N=87** | **≥60: No. (%);**  **N=74** | **p** |
| --- | --- | --- | --- | --- | --- | --- |
| Expense | 338 (68%) | 55 (60%)^a^ | 79 (64%) | 53 (61%) | 61 (82%)^a^ | .01 |
| Travel time | 286 (57%) | 55 (60%) | 65 (53%) | 50 (57%) | 40 (54%) | .75 |
| Discovering/searching for appropriate CME offerings | 143 (29%) | 30 (33%) | 25 (20%) | 25 (29%) | 21 (28%) | .02 |
| Not applicable to daily practice | 133 (27%) | 19 (21%) | 33 (27%) | 28 (32%) | 31 (36%) | .15 |
| Ability to easily track CME credits earned | 59 (12%) | 8 (9%) | 20 (16%) | 7 (8%) | 11 (15%) | .19 |

Responses were selected from a checklist, in response to the question: "What are the biggest gaps/pain points in obtaining CME offerings? [check all that apply]"

^a^ Subgroup responses are statistically significantly different from one another using Tukey's test (p<.01).

# e-Table 2. Sources of Information about CME Courses, by Age Group

| **Information Source** | **All: No. (%);**  **N=500** | **<40: No. (%); N=92** | **40-49: No. (%); N=123** | **50-59: No. (%); N=87** | **≥60: No. (%); N=74** | **p** |
| --- | --- | --- | --- | --- | --- | --- |
| Online | 348 (70%) | 68 (74%) | 88 (71%) | 53 (61%) | 45 (61%) | .12 |
| Direct email communication | 296 (59%) | 46 (50.0%)^a^ | 64 (52.0%)^a^ | 50 (57.5%) | 56 (75.7%)^a^ | .003 |
| Professional associations | 293 (59%) | 54 (58.7%) | 68 (55.3%) | 55 (63.2%) | 43 (58.1%) | .73 |
| Brochures via direct mail | 263 (53%) | 40 (43.8%)^a^ | 52 (42.3%)^a^ | 59 (67.8%)^a^ | 44 (59.5%) | <.001 |
| Journals | 260 (52%) | 45 (48.9%) | 64 (52.0%) | 46 (52.9%) | 44 (59.5%) | .60 |
| Peers/word of mouth | 234 (47%) | 57 (62.0%) | 56 (45.5%) | 40 (46.0%) | 28 (37.8%) | .013 |
| Healthcare organizations | 165 (33%) | 27 (29.3%) | 32 (26.0%)^a^ | 43 (49.4%)^a^ | 25 (33.8%) | .003 |
| Medical societies | 163 (33%) | 23 (25.0%) | 40 (32.5%) | 31 (35.6%) | 26 (35.1%) | .40 |
| Brochures via in-person handout | 104 (21%) | 18 (19.6%) | 22 (17.9%) | 24 (27.6%) | 20 (27.0%) | .25 |
| Doximity | 54 (11%) | 9 (9.8%) | 9 (7.3%) | 8 (9.2%) | 11 (14.9%) | .39 |
| Physician liaison | 27 (5%) | 5 (5.4%) | 6 (4.9%) | 5 (5.7%) | 5 (6.8%) | .96 |
| Online chat forums | 24 (5%) | 7 (7.6%) | 7 (5.7%) | 3 (3.4%) | 5 (6.8%) | .68 |
| A mentor | 21 (4%) | 9 (9.8%) | 4 (3.3%) | 6 (6.9%) | 1 (1.4%) | .06 |
| My supervisor | 15 (3%) | 5 (5.4%) | 5 (4.1%) | 1 (1.1%) | 2 (2.7%) | .44 |

Responses were selected from a checklist, in response to the question: "How do you find out about CME courses?"

^a^ Subgroup ratings are statistically significantly different from one another using Tukey's test (p<.01).

# e-Table 3. Time and monetary support available for CME, by Age Group

| **Domain** | **Response** | **All: No. (%);**  **N=500** | **<40: No. (%); N=92** | **40-49: No. (%); N=123** | **50-59: No. (%); N=87** | **≥60: No. (%); N=74** | **p** |
| --- | --- | --- | --- | --- | --- | --- | --- |
| Time allotment | 0 days | 59  (12%) | 13  (14%) | 12  (10%) | 12  (14%) | 11  (15%) | .50 |
|  | 1-6 days | 301 (60%) | 62  (67%) | 73  (59%) | 48  (55%) | 42  (57%) |  |
|  | ≥7 days | 121 (24%) | 16  (17%) | 30  (24%) | 23  (26%) | 19  (26%) |  |
|  | Irrelevant^a^ | 19 (4%) | 1  (1%) | 8  (7%) | 4  (5%) | 2  (3%) |  |
| Change in time allotment | Less | 79 (16%) | 13 (14%) | 20 (16%) | 17 (20%) | 10 (14%) | .49 |
|  | No change | 374 (75%) | 70 (76%) | 84 (68%) | 63 (72%) | 62 (84%) |  |
|  | More | 47 (70%) | 10 (11%) | 19 (6%) | 7 (8%) | 2 (3%) |  |
| Monetary allowance | <$1000/ none | 219 (44%) | 34 (37%) | 48 (39%) | 45 (52%) | 44 (59%) | .07 |
|  | $1,000 - $5,000 | 263 (53%) | 55 (60%) | 67 (54%) | 40 (46%) | 29 (39%) |  |
|  | >$5,000 | 18 (0%) | 3 (3%) | 8 (7%) | 2 (2%) | 1 (1%) |  |
| Change in monetary allowance | Less | 68  (15%) | 10  (11%) | 19  (15%) | 15  (17%) | 9  (12%) | .48 |
|  | No change | 358 (72%) | 75  (82%) | 91  (74%) | 57  (66%) | 54  (73%) |  |
|  | More | 45  (9%) | 10  (14%) | 12  (10%) | 5  (6%) | 5  (7%) |  |
|  | No support | 29 (5.8%) | 3  (3%) | 1  (0%) | 10  (12%) | 6  (8%) |  |

Questions asked about current time or monetary support from their practice for CME activities each year, and how the amount of time/monetary support had changed over the past 24 months.

^a^ Indicates response that the clinician would "Not [be] willing to be away from my practice"
